# Enabling High-Performance Hybrid Solid-State Batteries by Improving the Microstructure of Free-Standing LATP/LFP Composite Cathodes

**DOI:** 10.1021/acsami.3c18542

**Published:** 2024-04-01

**Authors:** Martin Ihrig, Enkhtsetseg Dashjav, Philipp Odenwald, Christian Dellen, Daniel Grüner, Jürgen Peter Gross, Thi Tuyet Hanh Nguyen, Yu-Hsing Lin, Walter Sebastian Scheld, Changhee Lee, Ruth Schwaiger, Abdelfattah Mahmoud, Jürgen Malzbender, Olivier Guillon, Sven Uhlenbruck, Martin Finsterbusch, Frank Tietz, Hsisheng Teng, Dina Fattakhova-Rohlfing

**Affiliations:** † Institute of Energy and Climate Research, IEK-1: Materials Synthesis and Processing, Forschungszentrum Jülich GmbH, 52425 Jülich, Germany; ‡ Department of Chemical Engineering, National Taiwan University of Science and Technology, No. 43, Keelung Rd., Section 4, Da’an Dist. Taipei City 106, Taiwan; § Faculty of Engineering and Center for Nanointegration Duisburg-Essen (CENIDE), Universität Duisburg-Essen, Lotharstraße 1, 47057 Duisburg, Germany; ∥ Institute of Energy and Climate Research, IEK-2: Microstructure and Properties Forschungszentrum Jülich GmbH, 52425 Jülich, Germany; ⊥ Graduate School of Engineering, Kyoto University, Nishikyo-ku, Kyoto 615-8510, Japan; # Department of Chemical Engineering, National Cheng Kung University, Tainan 70101, Taiwan; ∇ GREENMat, CESAM Research Unit, Institute of Chemistry B6, University of Liège, 4000 Liège, Belgium; ○ Hierarchical Green-Energy Materials (Hi-GEM) Research Center, National Cheng Kung University, Tainan 70101, Taiwan; ◆ Center of Applied Nanomedicine, National Cheng Kung University, Tainan 70101, Taiwan

**Keywords:** composite cathode, LATP, LFP, polymer–ceramic, all-solid-state Li battery, microstructure optimization, mechanical properties

## Abstract

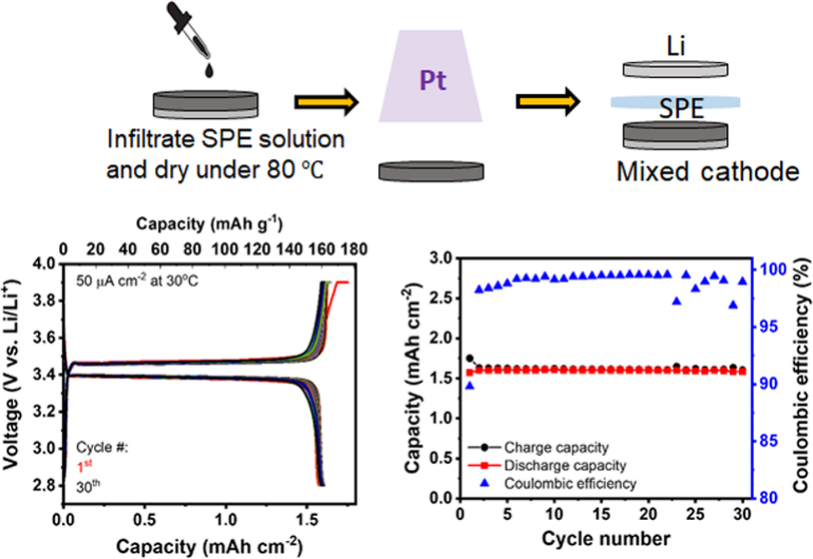

The phosphate lithium-ion conductor Li_1.5_Al_0.5_Ti_1.5_(PO_4_)_3_ (LATP) is an economically attractive solid electrolyte for the fabrication of safe and robust solid-state batteries, but high sintering temperatures pose a material engineering challenge for the fabrication of cell components. In particular, the high surface roughness of composite cathodes resulting from enhanced crystal growth is detrimental to their integration into cells with practical energy density. In this work, we demonstrate that efficient free-standing ceramic cathodes of LATP and LiFePO_4_ (LFP) can be produced by using a scalable tape casting process. This is achieved by adding 5 wt % of Li_2_WO_4_ (LWO) to the casting slurry and optimizing the fabrication process. LWO lowers the sintering temperature without affecting the phase composition of the materials, resulting in mechanically stable, electronically conductive, and free-standing cathodes with a smooth, homogeneous surface. The optimized cathode microstructure enables the deposition of a thin polymer separator attached to the Li metal anode to produce a cell with good volumetric and gravimetric energy densities of 289 Wh dm^–3^ and 180 Wh kg^–1^, respectively, on the cell level and Coulombic efficiency above 99% after 30 cycles at 30 °C.

## 1 Introduction

Solid-state batteries are one of the most intensively researched concepts for next-generation batteries, which promise to improve battery performance in terms of energy density, extended temperature range, and higher safety.^1−5^ In this context, ceramic solid electrolytes are among the attractive material systems that offer high intrinsic safety at the cell level due to their nonflammability.^6−8^ Among the various classes of ceramic electrolytes, NaSICON-type systems with the general formula Li_1+x_Al_*x*_Ti_2–*x*_(PO_4_)_3_ (0 < *x* < 0.5) (LATP) exhibit only a moderate ionic conductivity of up to 1 mS/cm at room temperature (RT), but they have high oxidation stability, high mechanical strength, and relatively low density compared to other ceramic electrolytes, which enables higher specific energy of battery cells.^9^ LATP is composed of abundant and inexpensive elements and can be produced on a large scale, making it technologically and economically attractive.

The use of LATP in various battery designs has been demonstrated by numerous groups, and the number of publications is steadily increasing.^10−21^ LATP has been successfully used in hybrid systems in combination with polymers to produce separators^22,23^ and as a solid electrolyte in solid-state cells in combination with inorganic cathode active materials (CAMs).^24,25^ Even some fully inorganic LATP-based solid-state battery concepts have already been commercialized.^12,26^

To achieve practical energy densities, solid-state batteries should contain thick cathode layers with a high CAM loading and a thin separator layer, ideally in combination with a high-energy anode such as lithium metal. In a predominantly ceramic cell design, fabrication of the cathode layer is the most critical step in terms of fundamental material properties. In addition to a high CAM loading, the cathode layer should have sufficiently high ionic and electronic conductivity to enable practical energy density at reasonable rates. It also needs to be fabricated in a scalable manner suitable for commercialization. One of the approaches being intensively pursued is the fabrication of free-standing (self-supporting) composite cathodes by tape casting. Tape casting is a well-established commercial technique that can continuously produce electrode layers with adjustable thickness.^27^ Several groups, including our group, have successfully used the tape casting technique to fabricate free-standing composite cathode layers consisting of a sintered percolating network of LATP and LiFePO_4_ (LFP) as the CAM. LATP forms a mechanically stable framework in the architecture of the composite cathode, which helps to reduce the volume changes of LFP during cycling (about 6%).^28^ In addition, the slurries are carburized during sintering in an inert atmosphere and form a percolating carbonaceous network required for electronic conductivity.^16,29^ The sintered tape-cast cathodes have shown promising performance in hybrid cells after infiltration with polymer electrolytes that increase ionic conductivity compared to single electrolytes.^11,30^

One of the major remaining challenges in the fabrication of composite cathodes is the high surface roughness of the sintered tapes with the formation of large crystals of LATP or LFP that are detrimental to optimized cell geometry. The rough surface prevents the deposition of thin separators, which are required for a high energy density. The protruding crystals do not allow continuous deposition of the separator layer or easily penetrate the separator and cause a short-circuit. A smooth, homogeneous surface of the inorganic cathodes is therefore very important for integration into cells with a practical energy density. Whereas surfaces of sintered pellets can easily be polished by using sandpaper, this is not possible for thin, free-standing tapes. Furthermore, any avoided processing step (such as polishing) is beneficial for scaling up the tape casting process for battery production.

The main reason for the surface roughness is crystal growth during sintering, which is required to connect the solid electrolyte and CAM particles. Attempts to minimize roughness by reducing the sintering temperature or sintering time usually fail due to the insufficient mechanical stability of the resulting cathodes. An alternative strategy for surface optimization is the addition of lithium tungstate (Li_2_WO_4_, LWO) to the casting slurry. LWO, a material with a low melting temperature of 742 °C, has already been recognized as a very efficient sintering aid for densifying pure LATP, lowering the sintering temperature by more than 100 °C.^31^ In this work, we show that LWO can also be added to the composite cathode, lowering the sintering temperature without affecting the phase composition of the LATP and LFP materials and resulting in mechanically stable, self-supporting cathodes with a smooth, homogeneous surface. The advantages of the optimized cathode microstructure are demonstrated in cell fabrication, which enables the deposition of a thin polymer separator layer without a supporting ceramic sheet^15^ and a cell assembly that operates with lithium metal anodes without short-circuiting.

## 2 Results and Discussion

Free-standing cathode tapes consisting of LFP as CAM and LATP as a solid electrolyte were cast onto a mylar foil from a slurry containing LFP and LATP powders, solvent, dispersant, binder, and plasticizer and dried at RT (see the Experimental Section and Methods and Figure S1 in the Supporting Information for more details), following a previously described procedure.^16^ In a modified procedure used in this work, 5 wt % of LWO was added to the slurry to optimize the morphology of the tapes after sintering, and the ratio of LFP and LATP was varied from 40:60 to 60:40 wt %. The resulting green tape was compressed at a mechanical pressure of 210 MPa to increase the relative density. Some of the green tapes were sintered without compression for comparison. The green tapes were annealed at temperatures between 700 and 800 °C for 1 h to sinter the ceramic powders. Due to the very low electronic conductivity of the LFP phase, a conductive additive such as carbon is required to increase the overall conductivity of the electrodes. This was achieved by sintering in an Ar atmosphere, which leads to carburization of the organic components and formation of a conductive graphite phase.

**Figure 1:**
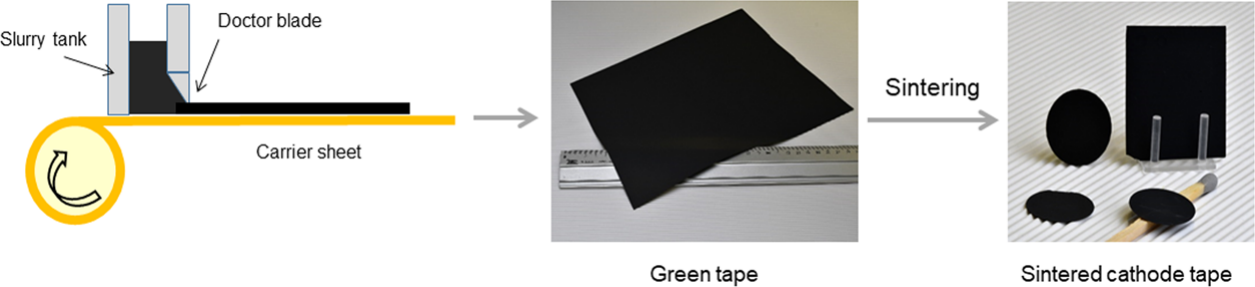
Schematic overview of the tape casting process and the optical images of the resulting green and sintered LFP–LATP/LWO cathode tapes.

The tapes containing LFP and LATP require a temperature of 800 °C to sinter the powders and obtain mechanically stable cathodes. By adding 5 wt % of LWO, the sintering temperature could be lowered by 100 °C, so that flat, black, and mechanically stable free-standing, less than 100 μm thick composite cathodes were obtained at a temperature of only 700 °C (Figure 1). In a reducing environment (Ar atmosphere and the presence of carbon-containing materials), LWO decomposes already below 700 °C forming WO_2_ and releasing Li ions, which activate the sintering process of LATP and LFP surfaces before the melting point of LWO.

### 2.1 Phase Composition and Microstructure of LFP–LATP/LWO Cathode Tapes

**Figure 2:**
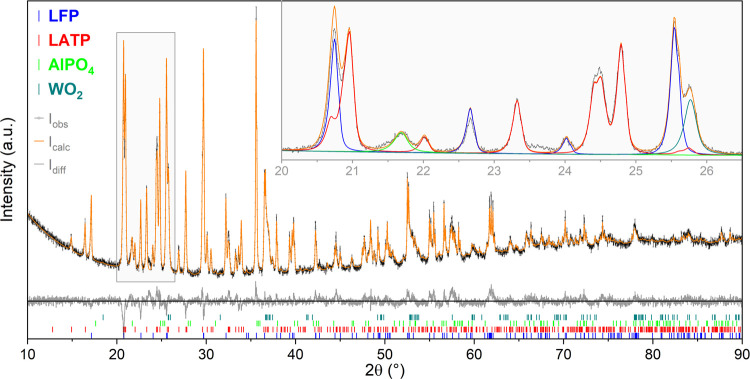
Phase analysis of the XRD pattern of the sintered LFP–LATP (60:40) composite with 5 wt % of LWO indicates the olivine-type LFP and orthorhombic LATP as main phases. Minority phases are AlPO_4_ and WO_2_. Contributions of individual phases with their calculated diffraction patterns are shown in the inset.

X-ray diffraction (XRD) analyses of the sintered cathodes prepared with 5 wt % of LWO showed similar phase composition compared to the already reported sample without LWO. LFP and LATP were found as the main phases. Small amounts of AlPO_4_ were found as a minority phase with additional WO_2_ as a second minority phase due to the addition of LWO. Careful analysis of the crystal structure of LATP showed that it was completely converted to the orthorhombic modification after sintering under Ar in all samples (with and without LWO). The conversion to the orthorhombic phase was previously reported for LATP sintered in a reducing atmosphere in the presence of carbon and an excess of Li_2_O.^32−34^ Therefore, the best agreement with the observed XRD pattern was obtained with a mixture of olivine-type LFP (*Pnma*, ICSD no. 15445), orthorhombic LATP (Pbna, ICSD no. 151919), monoclinic WO_2_ (C2_1_/c, ICSD no. 80829), and orthorhombic AlPO_4_ (*C*222_1_, ICSD no. 98378). The results of the phase analysis based on the Rietveld refinement of the XRD patterns are summarized in Table 1. Figure 2 shows the observed and calculated XRD patterns of the sintered LFP–LATP (60:40) composite with 5 wt % of LWO as an example. Further details on the phase analysis based on XRD data can be found in the Supporting Information, Table S1 (lattice parameters and refinement statistics for all samples) and Figure S2 (observed and calculated XRD patterns of LATP–LFP 60:40 without LWO).

**Table 1:** Phase Fractions of the LFP–LATP/LWO Composites Are Based on the Rietveld Refinement of the XRD Results

	amount [wt %]			
LFP/LATP + 5 wt % LWO	LFP	LATP	WO_2_	AlPO_4_
40:60	41	54	3	2
50:50	53	44	2	1
60:40	64	33	2	1
60:40 without **LWO**	65	33	0	2

**Figure 3:**
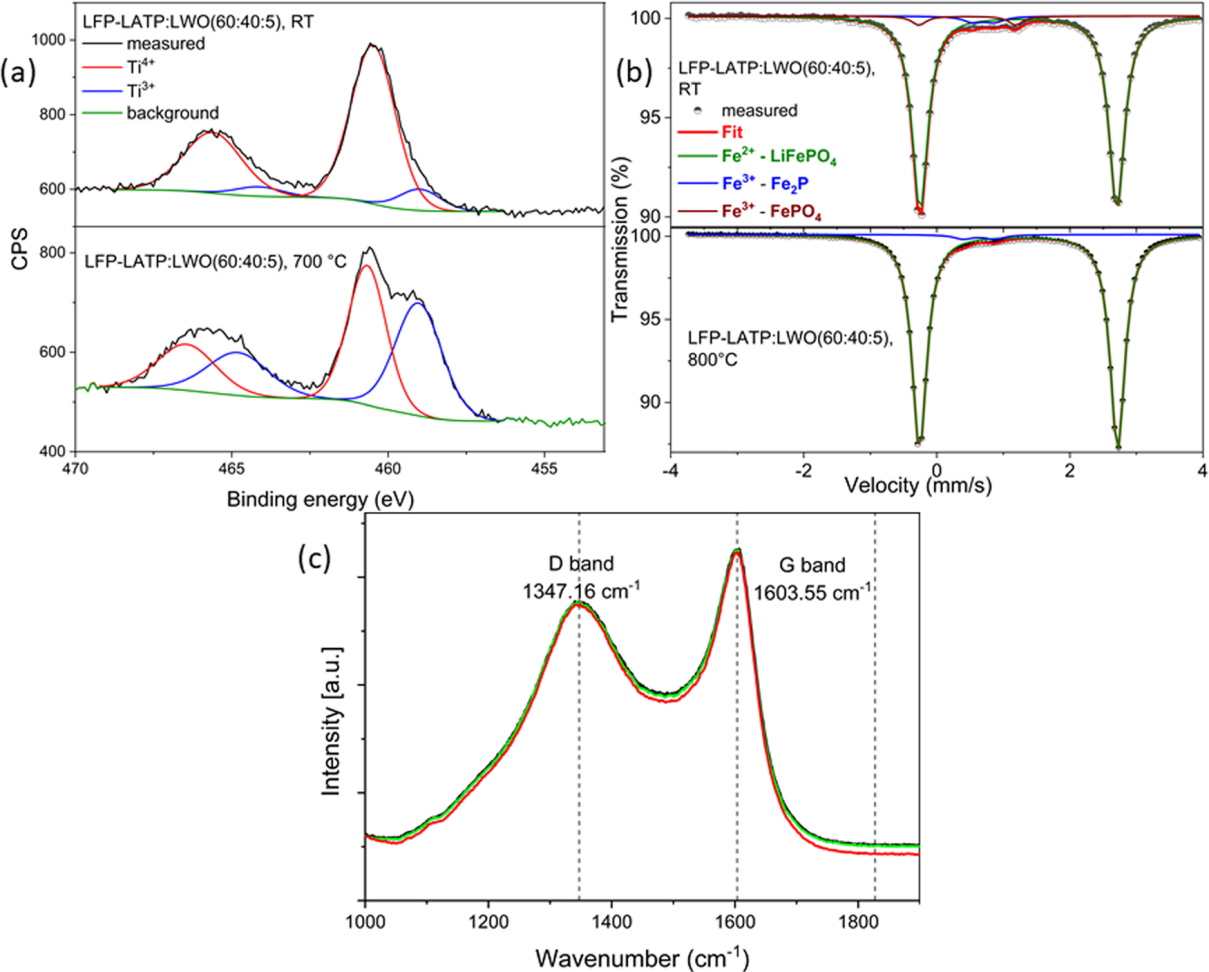
(a) Ex situ XP spectra of Ti in an LFP–LATP/LWO (60:40:5) composite measured at RT: as-cast cathode tape (top) and after sintering in Ar at 700 °C (bottom). (b) Mössbauer spectra recorded at RT for the as-cast cathode tape (top) and after sintering in Ar at 800 °C (bottom). (c) Raman spectra taken from three different positions on the LFP–LATP/LWO (60:40:5) cathode tape annealed at 700 °C in Ar, showing the D and G bands of amorphous and graphitic carbon, respectively.

Rhombohedral and orthorhombic LATP polymorphs can be easily distinguished by analyzing the oxidation state of titanium ions by using X-ray photoelectron spectroscopy (XPS) (Figure 3a,b). The rhombohedral LATP structure contains only Ti^4+^ ions (Figure S3), while the orthorhombic LATP is a mixed-valent compound Li_1.5+x_Al_0.5_(Ti_1.5–*x*_^4+^Ti_*x*_^3+^)(PO_4_)_3_ containing a mixture of Ti^3+^ and Ti^4+^ ions. The XPS spectrum of an LFP–LATP/LWO (60:40:5) tape without heat treatment shows peaks at 460 and 466 eV, corresponding to Ti 2p3 and Ti 2p1 states of Ti^4+^ ions, respectively, with a relative fraction of 88.8 atom % (Table 2), which explains the predominant presence of the rhombohedral LATP phase in the green tape (Figure 3a, top). 11.2 atom % of Ti^3+^ could indicate some structural defects around Ti^4+^ within LATP.

**Table 2:** Deconvoluted Areal Distribution of Ti^3+^ and Ti^4+^ in LFP–LATP/LWO (60:40:5) Composites by XPS. The values are normalized to 100%

temperature (°C)	25	600	650	700	750	800	850
Ti^3+^ (%)	11.2	34.9	41.5	49.7	44.3	38.7	32.4
Ti^4+^ (%)	88.8	65.1	58.5	50.3	55.7	61.3	67.6

After annealing at 700 °C in Ar, additional peaks appear at 458 and 464 eV (Figure 3a, bottom), indicating the presence of Ti^3+^. Hence, annealing in an inert atmosphere in the presence of carbon leads to the reduction of Ti^4+^ to Ti^3+^ in LATP, triggering the structural transition from rhombohedral to orthorhombic LATP. The highest relative fraction of 49.7% of Ti^3+^ ions was found for the sample annealed at 700 °C (Table 2). At higher annealing temperatures (*T* > 750 °C), the fraction of Ti^3+^ decreases again. This can be attributed to the evaporation of Li_2_O, which is required for the stabilization of the orthorhombic phase.

To investigate the valence changes in LFP as a function of sintering conditions, Mössbauer spectroscopy was used to analyze the oxidation state of Fe (Figure 3b). The spectra of LFP–LATP/LWO (60:40:5) before sintering were fitted with three doublet components, where the main component corresponds to 92 atom % Fe^2+^ and the second and third components correspond to 8 atom % Fe^3+^. The spectra of LFP–LATP/LWO annealed at 800 °C in Ar contain two peaks fitted with two doublets corresponding to 97 atom % Fe^2+^ and a small amount (3 atom %) Fe^3+^ (Figure 3b; hyperfine parameters are listed in Table S2). Two different types of Fe^3+^ signals in the spectrum of untreated LFP–LATP/LWO correspond to two different materials. An isomer shift δ of 0.69 mm/s is typical for Fe_2_P,^35^ with small amounts of Fe_2_P below the detection limit of XRD and XPS (Figures 2 and 3a,b). This impurity is also present in the sample after heat treatment at 800 °C with a slightly different isomer shift of 0.63 mm. The second Fe^3+^ signal with an isomer shift of 0.45 mm/s agrees very well with the spectra of distorted Li_*x*_FePO_4_ corresponding to the partially Li-deficient and oxidized LFP.^36^ The latter signal was no longer observed after heat treatment at 800 °C, suggesting that the distorted Li_*x*_FePO_4_ is converted to olivine-type LFP. No additional Fe^3+^ satellite peaks or changes in the intensity and shape of the Fe^2+^ peaks were observed in the spectrum of the heat-treated sample. Thus, the diffusion of Fe into LATP could be ruled out, as this would cause additional Mössbauer signals. The Mössbauer spectroscopy results agree well with the XRD analysis results (Figure 2), which show that the olivine LFP phase does not change after annealing and that the lattice parameters remain constant within the standard deviation for all samples before and after thermal treatment up to 800 °C.

Raman spectroscopy was used to characterize the state of the carbon that provides the pathways of electronic transport since neither LFP nor LATP exhibits sufficient electronic conductivity. Since the carbon was formed by carburizing the organic binder used for the tape casting process, it can be either amorphous or graphitic carbon.^37^ Amorphous carbon exhibits a Raman band known as the D band, while graphitic carbon exhibits a characteristic G band. In the measured Raman spectra of the LFP–LATP/LWO, both the D band at 1347 cm^–1^ and the G band at 1603 cm^–1^ are visible (Figure 3c). The homogeneity of the carbon polymorphs was tested at three different positions in the cross section of the annealed cathode tape. The degree of graphitization can be estimated from the ratio of the intensities of the D and G bands (*I*_D_/*I*_G_).^38^ The measured *I*_D_/*I*_G_ intensity ratio of the carbon is 0.85 on average, indicating partial graphitization toward an ordered carbon structure and thus an electronic percolation network throughout the cathode tape. The high electronic conductivity of the sintered LFP–LATP/LWO cathode composite was confirmed by four-point conductivity measurements, which yielded a specific resistance of 6 Ω·cm.

**Figure 4:**
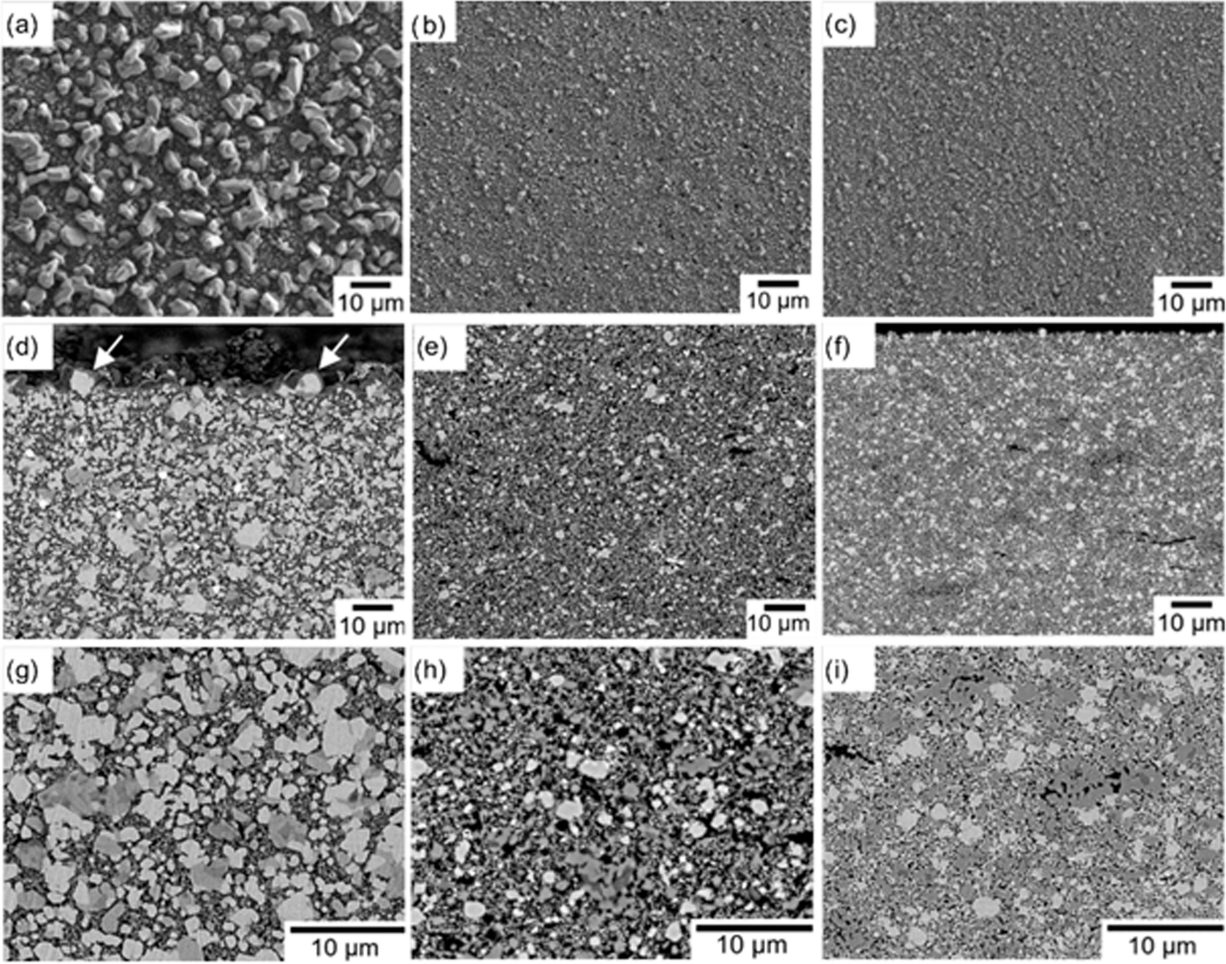
SEM micrographs of the surface ((a–c) secondary electron images) and polished cross sections ((d–i) backscattered electron images) of sintered LFP–LATP (60:40) without LWO (a, d, g) and noncompressed (b, e, h) and compressed (c, f, i) LFP–LATP/LWO (60:40:5). Arrows in panel (d) indicate large, protruding grains at the surface.

The above results confirm that the addition of 5 wt % LWO to the slurry has no effect on the phase composition of the sintered cathode tapes. However, significant differences were observed in the microstructure and surface roughness of the cathodes prepared with and without LWO. As shown in the scanning electron microscopy (SEM) images, the sintered LFP–LATP (60:40) cathodes without LWO contain LATP and LFP particles larger than 10 μm (Figure 4a,d,g) throughout the samples. Large protruding LATP and LFP grains were also found on the surface (Figure 4a), resulting in significant surface roughness. The protruding grains are detrimental to the battery assembly because they can puncture the separator and cause short-circuits. In contrast, both the bulk and surface microstructure of noncompressed, LWO-containing samples consist of much smaller LATP and LFP grains up to 2 μm in size (Figure 4b,e,h) and even smaller grains below 0.2 μm that were assigned to LFP and LWO/WO_2_, respectively, by energy-dispersive X-ray spectroscopy (EDX) analysis. LWO acts as a sintering aid and enables lower sintering temperatures for the compaction of LFP–LATP. Without the addition of LWO, LFP–LATP tapes must be sintered at temperatures of 800 °C or higher to establish contact between the particles; however, at these temperatures, the particles begin to grow uncontrollably due to Ostwald ripening. By the use of LWO as a sintering aid, the contact between the particles could already be established at a temperature of 700 °C. At this temperature, particle growth is still very slow and can be well controlled. This grain morphology resulted in a smooth, homogeneous surface of LFP–LATP–LWO cathodes (Figure 4b,c). It should be noted that EDX cannot distinguish between LWO and WO_2_ because the energy of the X-ray emission line of Li is too low to be detected. The W-rich phase was identified as WO_2_ by XRD, as described above. A small amount of AlPO_4_ was present as a minority phase in cathodes with and without LWO, consistent with the XRD results.

The addition of LWO thus improves the surface morphology of the composite cathodes but results in a lower density of the samples due to the lower sintering temperature. To improve the densification behavior, the green LFP–LATP–LWO tapes were mechanically compressed at a pressure of 210 MPa for 2 min before sintering. The grain size distribution and surface structure of these compressed LWO-containing LATP–LFP cathodes are very similar to those of the nonlaminated cathodes, but the density is significantly increased, resulting in improved mechanical stability (Figure 4c,f,i; see also Table 3).

**Table 3:** Relative Porosity (*p*_rel_), Specimen Thickness, Weibull Modulus (*m*), and Characteristic Strength (σ_char_) with the Upper and Lower Limits of the Confidence Interval of Free Sintered Tapes and Tapes Compressed Prior to Sintering

sintered LFP–LATP/LWO	*p*_rel_ [%]	thickness [μm]	*m* [−]	σ_char_ [MPa]
40:60:5	49.5 ± 0,5	103 ± 33	3.1_2.1_^3.9^	40_35_^46^
60:40:5	51.0 ± 0.7	98 ± 10	3.7_2.6_^4.7^	27_24_^30^
50:50:5	47.8 ± 0.2	108 ± 7.1	4.4_3.1_^5.6^	35_32_^39^
60:40:0	39.0 ± 0.8	-	-	-
Compressed and Sintered				
40:60:5	32.3 ± 0.8	60 ± 2.0	6.3_4.7_^7.7^	61_58_^64^
60:40:5	32.1 ± 1.4	83 ± 4.4	10.2_7.7_^12.5^	69_67_^71^
50:50:5	31.8 ± 1.4	87 ± 0.9	6.6_4.9_^8.0^	64_61_^67^

**Figure 5:**
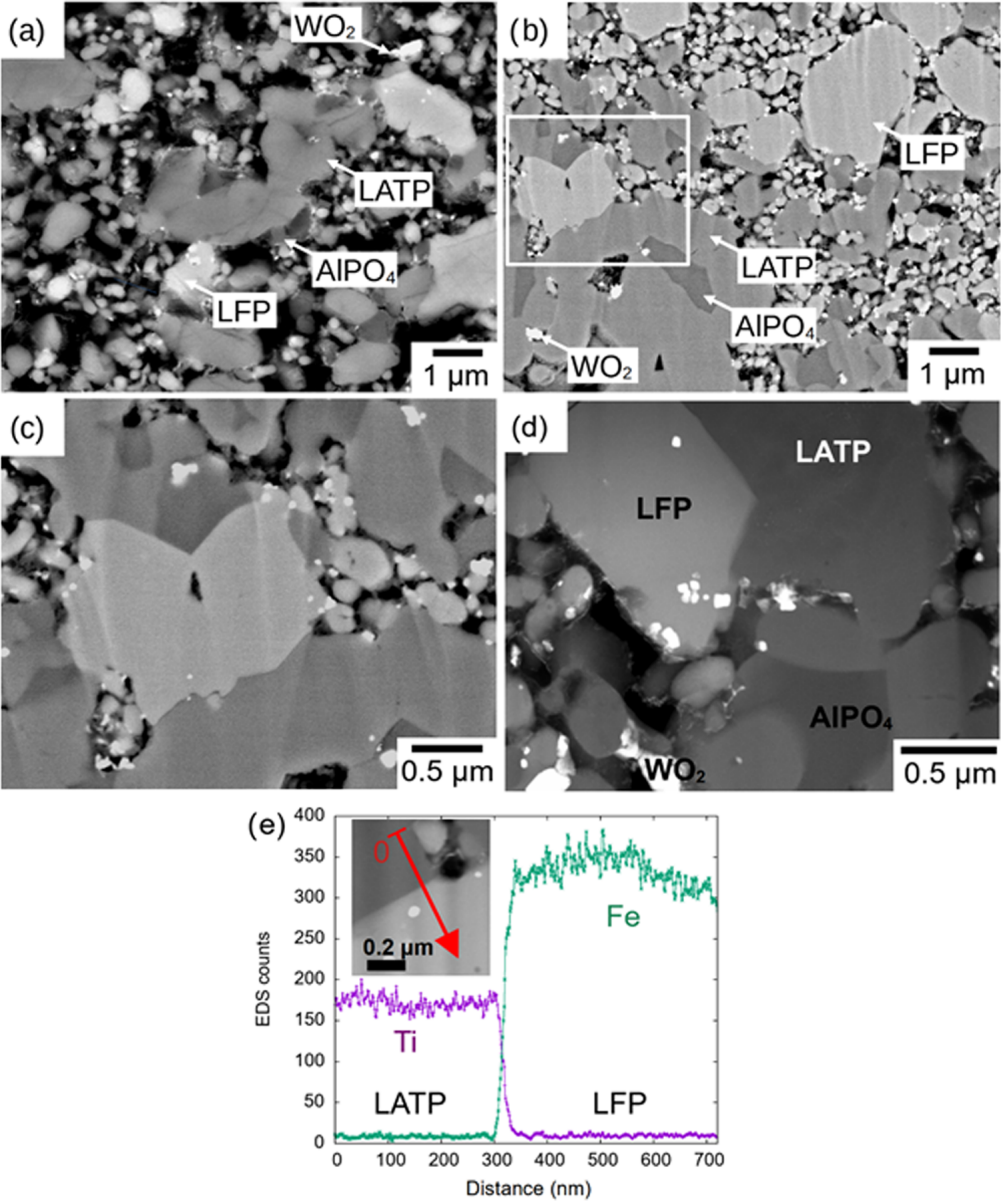
Representative SEM micrographs of ion-polished (a) noncompressed and (b) compressed and sintered LFP–LATP/LWO with LFP–LATP ratio 60:40 after sintering at 700 °C. Four individual phases are distinguishable by EDX measurements identified as AlPO_4_, LFP, LATP, and WO_2_. Panel (c) shows details of the microstructure in the region indicated by the box in panel (b), revealing sharp interfaces between phases. (d) STEM-HAADF image of the sintered LFP–LATP/LWO composite indicating clearly distinct phases. (e) EDX line scan of Ti and Fe across an LATP–LFP interface. The location of the line scan is shown in the inset. The Fe and Ti profiles indicate a sharp diffusion-free LFP–LATP interface.

Backscattered electron images (Figure 5a–c) and EDX mappings at low excitation energy (3 keV, Figure S4a; for comparison, EDX mappings of LATP–LFP without LWO are shown in Figure S4b) of the noncompressed and compressed LWO-containing cathodes show sharp and well-defined boundaries between the LFP and LATP particles after sintering. These results were also confirmed by a more detailed analysis of the LWO-containing cathode by using transmission electron microscopy (TEM). Scanning transmission electron microscopy combined with high-angle annular dark-field (STEM-HAADF) images show LATP and LFP as major phases with no evidence of interfacial diffusion, carbon in partially filled pores, and WO_2_ and AlPO_4_ as minor phases that can be distinguished by a clear gray contrast (Figure 5d). TEM–EDX line scans were acquired to evaluate the interfacial diffusion between the LFP and LATP phases. Due to the porosity of the LFP–LATP composites, a rather thick TEM lamella had to be fabricated, which exhibited a pronounced thickness variation. Only a few LFP–LATP interfaces were visible. Most of them were inclined to the incident beam, but a few interfaces were aligned with the beam. An analysis of the signals from Ti (as a marker for LATP) and Fe (as a marker for LFP) in a line scan across an aligned LFP–LATP interface showed sharp steps (*d* = 10 nm) and thus a diffusion-free interface (Figure 5e). The sharp LFP–LATP interfaces observed in the LWO-containing cathodes are different from previously reported LFP–LATP cathodes, where interdiffusion layers in the range of 40–100 nm containing Ti and Fe were commonly observed.^21^

### 2.2 Mechanical Properties of Sintered LFP–LATP/LWO Cathode Tapes

The fracture stresses and their variability, determined using the so-called Weibull modulus, are essential for estimating the reliability of the LFP–LATP/LWO composite cathodes. The fracture stress of the LATP framework is critical for its resilience and thus for compensating for the volume changes of LFP during cycling.

The estimated porosities of the materials obtained from the image analysis are shown in Table 3. They are about 50% for noncompressed and about 33–32% for compressed tapes, with a very small variation. These data confirm our observation above that the compression of the green tapes improves densification.

The results of the statistical analysis of the bending test for at least 30 specimens are given in Table 3. Specimen thickness scatters considerably for the noncompressed tapes, while the thickness variation is much smaller for the compressed specimens. The noncompressed materials with different LATP/LFP ratios have similar Weibull moduli and characteristic strengths, indicating a small influence of this parameter on the mechanical properties. The Weibull moduli and characteristic strength increase by almost a factor of 2 after compression. The lower Weibull moduli of the noncompressed materials could be due to both the large thickness variation and the lower relative density of the test specimens.

Similar to the Weibull modulus, the characteristic strength of the specimens depends mainly on the relative densities of the specimens, as can be seen in Figure S5a, masking any potential influence of the different LFP/LATP ratios. The observations are supported by the fractographic analysis of the specimens, which shows that large pores seem to be the main cause of failure in the bending tests (Figure S5b).

### 2.3 Performance of the LFP–LATP/LWO Cathode Tapes in a Full Cell

For cell assembly, disks were punched from the LFP–LATP/LWO (60:40:5) cathode green tape, compressed at 210 MPa, and sintered at 700 °C to form free-standing cathodes with a thickness of about 75 μm and a diameter of about 12 mm. The cathodes were first infiltrated with a solution of interpenetrating polymer network electrolyte (IPNE), a combination of cross-linked poly(ethylene oxide)-based and poly(vinylidene fluoride)-based solid polymer electrolytes, and dried at 80 °C to solidify the IPNE and completely remove the residual solvent. The same solution was also used to prepare a free-standing IPNE membrane with a thickness of 70 ± 5 μm.^39^ A Pt contact was sputtered onto one side of the IPNE-impregnated cathode disks as a current collector. Then, the IPNE membrane and a Li metal foil were mechanically attached to the other side of the cathode. The full cell was sealed in a CR2032 coin cell for electrochemical measurements.

**Figure 6:**
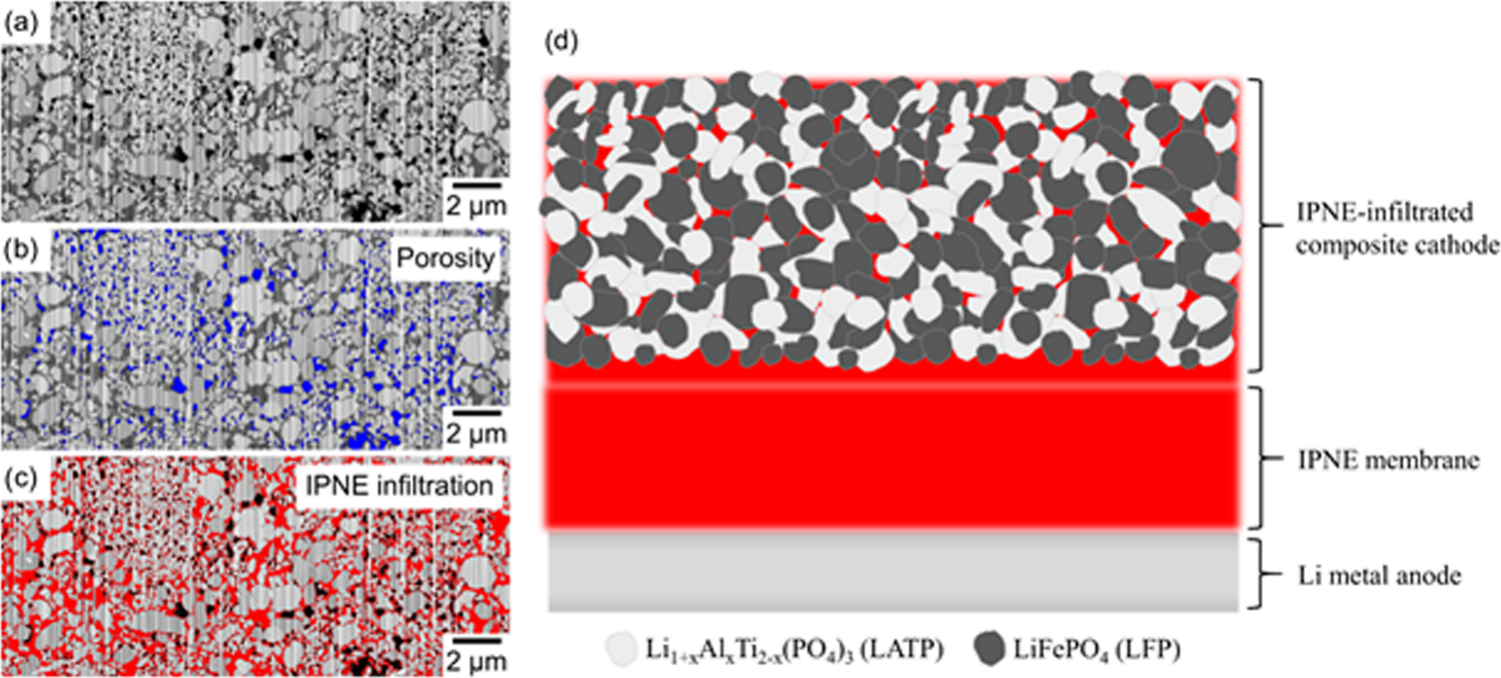
(a) FIB-SEM image of the infiltrated LFP–LATP/LWO (60:40:5) composite cathode, (b) highlighted porosity in blue of the same area, (c) highlighted IPNE polymer in red of the same area, and (d) schematic illustration of the full cell design with the thickness of the components.

The polymer-impregnated composite cathode tapes were analyzed by focused ion beam scanning electron microscopy (FIB-SEM) in order to obtain information about the morphology of the cathode and the degree of polymer infiltration. The FIB-SEM image (Figure 6) shows a uniform distribution of LFP particles (bright contrast), LATP particles (gray contrast), and small pores (black contrast, colored blue in Figure 6b for better visualization) throughout the thickness of the sintered LFP–LATP/LWO tapes. In addition to empty pores, the IPNE polymer can also be seen as a dark gray contrast (colored red in Figure 6c for better visualization) across the entire cathode thickness, showing that the IPNE polymer is evenly distributed in the cathode tape. Image analysis shows that the area occupied by the empty and polymer-filled pores accounts for 7.25 and 18.2% of the cathode cross-sectional area, respectively, so the total area occupied by empty and filled pores is 25.45%. Based on the areal fractions obtained from the FIB-SEM analysis and the known material density, the volume fractions of different components in the infiltrated composite cathodes were calculated (Table 4).

**Table 4:** Volume Fractions of the Infiltrated Composite Cathode (Figure 6a)

material	LFP	LATP	IPNE	porosity	carbon	WO_2_
volume fraction, %	37.6	30.9	18.2	7.3	3.3	2.7

**Figure 7:**
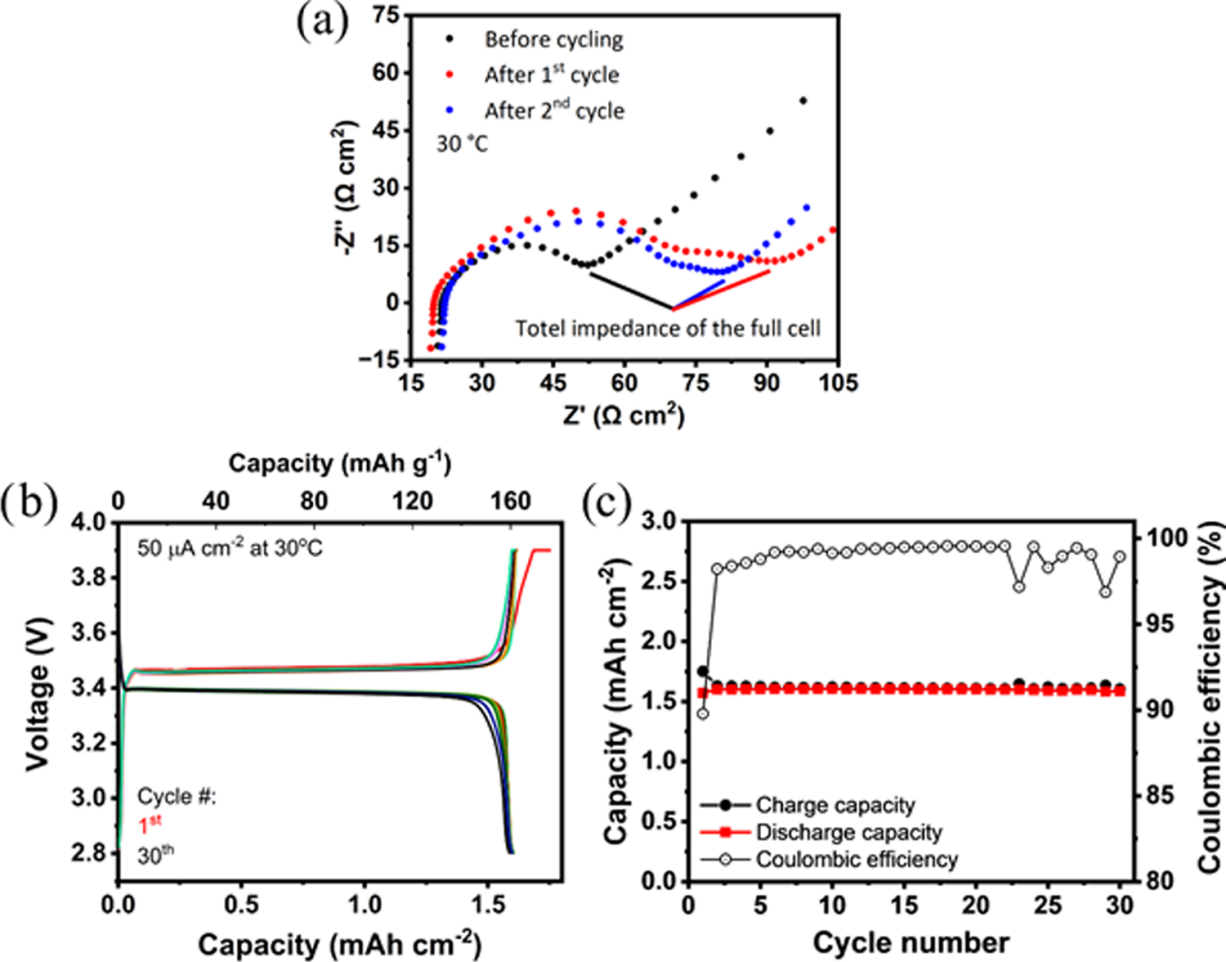
(a) EIS measurements of the cell LFP–LATP/LWO (60:40:5)-IPNE|IPNE|Li at 30 °C during the initial cycles with an LFP loading of 10.14 mg cm^–2^ (total composite cathode weight: 16.8 mg cm^–2^). In panel (b), the cycling behavior and (c) the areal capacity and Coulombic efficiency are shown.

The IPNE-infiltrated cathode tape can be easily attached to the IPNE membrane and then to the Li metal foil. This enables simple and quick assembly of the full cell, which is shown schematically in Figure 6d. The Nyquist plots of the electrochemical impedance spectra (EIS) of the as-assembled cells show only a compressed semicircle (Figure 7a). It can be assumed that the semicircle contains contributions from charge-transfer processes at different interfaces in the cell (e.g., LFP|LFP, LFP|LATP, LFP|polymer, LATP| polymer, and LATP|LATP and polymer|Li metal), which cannot be separated individually with sufficient accuracy at 30 °C (Figure 7a). Therefore, only the total impedance of the cell was determined, which is given by the intersection of the semicircle with the *x* axis at low frequencies. The total impedance of IPNE-infiltrated LFP–LATP/LWO (60:40:5)|IPNE|Li cell before cycling is about 77 Ω·cm^2^ at 30 °C, which is very low compared to typical values of other reported solid-state batteries and is even comparable to state-of-the-art commercial Li-ion batteries.^40^ The low total impedance and thus high conductivity even at 30 °C demonstrate the promising aspects of the presented IPNE-infiltrated LFP–LATP/LWO (60:40:5) composite cathode system. Within the first cycle, the total impedance increases slightly to 130 Ω·cm^2^ at 30 °C. This is accompanied by the formation of a new semicircle in the midfrequency range, which can be attributed to the increased interfacial resistance between the IPNE polymer and the LATP and/or LFP. While the infiltration of the IPNE polymer into the LFP–LATP/LWO composite cathode should lead to physical contact between the IPNE polymer and the LATP and/or LFP, it is possible that the transport of Li ions across the interface will result in changes of the conductivity of this interface or formation of a secondary phase.^41,42^ Interfacial conditioning occurs during the first cycle, as the total impedance of the cell increases with the formation of another semicircle. It is possible that the formation of the interface involves electrochemical reactions, as the first charge has a higher areal capacity than the subsequent charges (Figure 7b). On the one hand, the total impedance after the first charge is constant, and the “first discharge voltage” during subsequent cycles is comparable, suggesting that an irreversible interface formation process occurs during the first charging cycle. In particular, the results in Figure 7b,c indicate that these EIS changes during the first cycle have no negative impact on the electrochemical properties of this system, which shows stable cycling behavior from the second cycle onward. This observation is similar to the observations of Pervez et al. and Zhang et al.^41,42^ Pervez et al. observed that the interface between a solid electrolyte and an ionic liquid-based electrolyte is formed during the first cycles, which is then stable during the following cycles and ensures good electrochemical performance.^41^ Zhang et al. studied a polymer-in-ceramic-based electrolyte and reported that the conditioning of the interface during the first cycles is necessary for Li-ion transfer from the polymer to the ceramic electrolyte at 30 °C.^42^ Conditioning occurs within the first few cycles and leads to slightly increased charge capacities due to irreversible electrochemical reactions that occur during the formation of the polymer–ceramic interface. Thereafter, the interface is stable and enables stable cycling of the full cell. We assume that the interface between the IPNE polymer and the LFP and/or LATP forms in a similar manner and that their properties are comparable.

The galvanostatic curves of IPNE-infiltrated LFP–LATP/LWO (60:40:5)|IPNE|Li cell (Figure 7b) show well-defined plateaus at potentials of about 3.45 and 3.35 V vs Li/Li^+^ during charge and discharge, respectively. These plateaus are typical for LFP and are followed by a sharp voltage rise and fall at the end of the charge and discharge cycles, respectively. According to the changes in the EIS after the first cycle, a slightly higher capacity of 0.2 mAh or 10% of the total charge is measured compared with subsequent cycles. Moreover, the voltage rise at the end of the first charge cycle is less sharp than in the subsequent cycles, possibly due to previously described conditioning (or formation) processes of the polymer|LATP or polymer|LFP interfaces,^40,41^ which was also described in our previous work on hybrid polymer–ceramic cells.^16^ However, the conditioning accounts for only a small fraction of the overall charge. This means that a majority of highly conductive interfaces are already formed by the infiltration of the IPNE polymer into the LFP–LATP/LWO composite cathode.

With the exception of the first cycle, all cycles from the seventh to at least the 23rd cycle achieve a high Coulombic efficiency of over 99%. Stable cycling behavior and constant capacity were achieved for at least 30 cycles (Figure 7c). After 30 cycles, the measurements were interrupted for system maintenance, although no signs of degradation or capacity fading were observed, suggesting that the cycling stability of the cells could be much higher. The areal capacity during the first 30 charge/discharge cycles is 1.65 mAh cm^–2^, which is comparable to the value reported for commercial liquid electrolyte-based Li-ion batteries.^43^ Based on the LFP loading of 10.14 mg cm^–2^ in the LFP–LATP composite cathode, the specific capacity is 163 mAh g^–1^, with a utilization of 96% of the entire LFP (theoretical capacity of LFP: 170 mAh g^–1^^44^).

Compared to other solid-state Li batteries with LFP as CAM, the presented composite cathodes showed excellent capacity, Coulombic efficiency, cycling stability, and LFP utilization in the positive electrode (Table 5). Surprisingly, the IPNE-impregnated sintered cathodes containing LATP showed better performance than the cathodes containing only LFP and polymer solid electrolytes in terms of higher LFP loading, higher areal capacity, and lower operating temperature, despite the rhombohedral to orthorhombic LATP phase transformation described above. The reasons for the improved performance of sintered LATP-containing hybrid cathodes and the fundamental contribution of LATP to the overall conductivity of the cathode are still unknown and should be investigated in the future. However, it is possible that the increased electronic conductivity of the orthorhombic LATP phase overcompensates the decrease in ionic conductivity and thus accounts for the higher overall conductivity of the composite cathode.

**Table 5:** LFP Loading and Storage Capacity of LFP and Polymer- or LATP Electrolyte-Based Composite Cathodes

					cathode capacity		
cathode	LFP loading [mg cm^–2^]	current density [μA cm^–2^]	cycles	*T* [°C]	[mAh g^–1^]	[mAh cm^–2^]	refs
LFP–PVdF	1.2	10	100	60	150	0.18	(45)
LFP–PEO	∼5.0	170	100	65	130	0.65	(46)
LFP–PEO	3.0–5.0	100	50	60	140	0.42–0.70	(47)
LFP–PEO	3.3	185	210	60	155	0.53	(48)
LFP–C-PVdF	2.5	8	5	25	148	0.37	(41)
LFP–PEO	3.2	50	50	60	136	0.42	(18)
LFP/LATP–MEEP	19.6	50	34	60	165	3.20	(16)
LFP–C-PVdF	∼2.0	100	120	55	160	0.32	(49)
LFP–C-PVdF	4.7	85	100	60	155	0.73	(50)
LFP–C-PEO	5.0	160	100	65	140	0.70	(51)
LFP–C-PEO	3.0–5.0	100	150	40	120	0.36–0.60	(52)
LFP–C-PEO	1.3	35	100	60	139	0.18	(53)
LFP–C-PEO–PvDF	5.0	425	1000	60	118	0.59	(54)
LFP–C-PVdF	1.5	15	130	30	157	0.24	(42)
LFP–C-PVdF	2.0	15	20	50	118.3	0.24	(55)
LFP–C-PVdF	2.5	50	300	60	138.8	0.35	(56)
LFP–LATP/LWO– IPNE	10.14	50	30	30	163	1.65	this work

The only comparable performance of LFP-based hybrid electrodes was obtained with LFP/LATP–MEEP cathodes infiltrated with MEEP polymer reported by us earlier^16^ (Table 5), for which an even higher areal capacity of 3.20 mAh cm^–2^ was achieved using thicker tapes with higher LFP loading. However, it should be noted that this performance was also obtained at a higher temperature of 60 °C compared to that at 30 °C in this work. More importantly, due to the high surface roughness of the tape-cast cathodes described by Ihrig et al.,^16^ the use of a thin polymer separator was not possible and a thick ceramic LLZO separator had to be placed between the cathode tape and the Li metal anode to prevent short-circuits.

The advantages of the optimized microstructure and the smooth surface of the ceramic cathode layers described in the current work enable the use of thin ceramic separators in combination with Li metal anodes without short-circuiting, which significantly reduces the weight of the passive cell elements and increases the energy density at the cell level, opening up new perspectives for cell design. For the full cell described in this work with an ∼75 μm thick composite cathode, an ∼70 μm thick IPNE membrane separator, and an ∼50 μm thick Li metal anode (with a total cell thickness of 195 μm), a volumetric energy density of ∼290 Wh dm^–3^ and a gravimetric energy density of 180 Wh kg^–1^ were calculated. To the best of our knowledge, this is the first time that the energy density of cells with sintered ceramic cathodes has been specified for the entire cell and not just for the cathode level. Even for the nonoptimized cell, the values obtained are comparable to the energy density of state-of-the-art LFP-based LIBs with liquid electrolytes. It is expected that the energy density at the cell level can be increased by increasing the thickness of the composite cathodes, using high-voltage olivine cathodes (such as LiCoPO_4_) and optimizing the particle size, relative fraction, and distribution of CAM and LATP powders in the cathode tapes.

## 3 Conclusions

The sintering behavior and microstructure of ceramic LFP–LATP composite cathodes were greatly improved using LWO as a sintering aid. The additive enabled a lower sintering temperature and, more importantly, resulted in a smooth surface of the tape-cast composite cathodes suitable for deposition of thin separators. Sintering in an Ar atmosphere resulted in the formation of a carbon network with a significant amount of graphitic carbon, ensuring high electronic conductivity of the composite cathode.

Possible reactions between the different materials were investigated by SEM/TEM, XRD, XPS, and Mössbauer spectroscopy. XRD showed the presence of a stable olivine LFP phase and an orthorhombic mixed-valent Li_1.5+*x*_Al_0.5_Ti_1.5–*x*_^4+^Ti_x_^3+^ (PO_4_)_3_ as the main phases. The presence of Ti^3+^ was confirmed by XPS analysis, while Mössbauer spectroscopy excluded the presence of Fe atoms in LATP and thus a possible ionic interdiffusion during sintering. Moreover, SEM and TEM investigations revealed sharp LATP/LFP interfaces without Ti/Fe interdiffusion.

The resulting microstructure and properties of the LFP–LATP/LWO-based composite cathodes are ideal for infiltration with the IPNE electrolyte and the assembly of a battery with a thin polymer separator and a Li metal anode. The combination of polymer and ceramic components resulted in well-functioning solid-state Li batteries with a high areal capacity of about 1.65 mAh cm^–2^, nearly complete utilization of LFP (96%), corresponding to volumetric and gravimetric energy densities of 289 Wh dm^–3^ and 180 Wh kg^–1^, respectively, on the cell level and good cycling stability for at least 30 cycles at 30 °C with a high Coulombic efficiency of about 99%.

## 4 Experimental Section and Methods

### 4.1 Tape Casting of the LFP–LATP Composite Cathodes

The preparation of LFP–LATP composite cathodes is described in detail in our previous work.^16^ The LFP (*D*_50_ = 0.5 μm) used here was provided by Johnson Matthey GmbH, and the LATP was synthesized in-house as described elsewhere.^31^ Additionally, we added 5 wt % of LWO to the slurry based on the previous work,^30^ where the relationship between sintering behavior, densification, and ionic conductivity of LATP with LWO additives between 3 and 9 wt % was investigated. On this basis, the addition of 5 wt % of LWO is chosen, which resulted in the highest density of LATP tapes. Microstructural analyses showed that the addition of LWO hinders the crystal growth of the LATP and LFP particles, which is important for the cycling behavior of the composite. Punched disks of green tapes (diameter of 13 mm) were sintered on a quartz plate with a slow heating rate of 1 °C min^–1^ up to 500 °C for the gentle release of organics from the disk followed by further heating up to 700–800 °C at a rate of 5 °C min^–1^ and a dwell time of 1 h at a maximum temperature in an Ar atmosphere. During sintering, the disk shrank to 12 mm in diameter. Prior to sintering, the green disks were compressed with an applied mechanical pressure of 210 MPa to obtain a higher green density. Some of the disks were sintered without this compression step for comparison.

### 4.2 Phase Analysis

The crystallographic properties of the samples were analyzed by XRD measurements at RT. For this purpose, an EMPYREAN diffractometer (Malvern Panalytical) with a copper X-ray tube was used, operated at an accelerating voltage of 40 kV and 40 current of 40 mA XRD patterns were collected in the 2Θ range from 5 to 90° with a step size of 0.01° and a 0.5 s time per step. The XRD patterns were analyzed using TOPAS software (Bruker AXS GmbH). Crystal structure data necessary for the analysis were taken from the ICSD database.

The XPS measurements were carried out using a Phi5000 Versa ProbeII (ULVAC-Phi Inc.) spectrometer. The monochromatic Al Kα excitation was 1.486 keV, and an X-ray beam size was 200 μm^2^, with a 50 W, 15 kV power setting used for the measurements. A freshly prepared pellet (LFP–LATP/LWO (60:40:5)) was dried in a vacuum oven to avoid air and moisture being transferred to the XP spectrometer. XP spectra were recorded at room temperature first without any heat treatment and then heated to 600, 700, and 800 °C. The heating rate was 0.2 °C s^–1^, and the sample was held at a high temperature for 0.5 h before it was then cooled down to room temperature. After each heat treatment, XP spectra were recorded at room temperature. Survey scans were collected by using a pass energy of 187.5 eV (0.8 eV steps, 100 ms/step). High-resolution scans were obtained with a pass energy of 23.5 eV (0.1 eV steps, 100 ms/step). Each XP spectrum was charge-corrected to the binding energy by setting the C 1s peak to 284.5 eV. Atomic percentage values and elemental ratios were calculated from the peak-area ratios after correction with the experimentally determined sensitivity factors, being reliable within ±10%.

^57^Fe Mössbauer spectra were obtained by using a constant-acceleration spectrometer with a ^57^Co(Rh) source at room temperature for two pellets of LFP–LATP/LWO (60:40:5). The first pellet was just a mixture of powder without heat treatment, and the second pellet was heat-treated at 800 °C in an Ar atmosphere for 1 h. The Mössbauer spectral absorbers were prepared with ∼40 mg cm^–2^ of the material mixed with boron nitride. The spectrometer was calibrated at room temperature with α-Fe foil. The measurements were carried out in the velocity ranges of ±4 mm/s with optimal energy resolution at room temperature. The Mössbauer spectra were fitted with three Lorentzian doublets using the Fullham program.

Raman spectra were collected with an inVia Raman Microscope (Renishaw) equipped with a 532 nm laser, a grating with 1800 lines/mm, and a CCD detector. The carbon distribution in the LATP–LFP composite was analyzed on a polished cross-sectional sample at multiple spots. At each spot, a mapping with an area of 6 × 6 μm^2^ and with a step size of around 0.21 μm was performed, leading to a total of 784 spectra per map. The subsequent data processing was performed with WiRE software (WiRE 5.2). First, a cosmic ray removal was performed, and afterward, the spectra of each mapped area were averaged to produce better statistics of the peak intensities of the D and G bands of carbon.

### 4.3 Microstructural Analysis by Scanning and Transmission Electron Microscopies

The sintered specimens were embedded in anhydrous epoxy resin for microstructural characterization. The cross-sectional surface of the embedded specimens was hand-finished on a Saphir 360 rotary grinder (ATM Qness GmbH) at 250 rpm using 400–4000 grit SiC abrasive paper. Fine polishing was performed with a Minimet 1000 polisher (Buehler). Anhydrous diamond suspensions with grit sizes of 6, 3, and 1 μm were used as polishing media. Final polishing was carried out with anhydrous colloidal silica (LUDOX AM, Sigma-Aldrich) with a particle size of 0.2 μm.

Polished samples were analyzed by using a Zeiss Merlin scanning electron microscope. Porosity was determined by image analysis using the ImageJ software (NIH) based on at least 6 SEM images per sample. The pores were identified by grayscale matching. For high-resolution SEM studies, polished cross sections of selected samples were prepared by Ar ion milling (SM09010 Cross Section Polisher, Jeol).

EDX spectra were acquired using X-Max Extreme and X-Max 80 detectors (Oxford Instruments). The collected data were analyzed by using the associated Aztec software.

A more detailed microstructural analysis was performed by TEM. The TEM lamella was prepared from a polished cross section of the composite cathode material by focused ion beam milling using a Zeiss Auriga dual-beam instrument. The lamella was fixed on a copper half-ring and investigated using a Zeiss Libra TEM operating at 200 kV in scanning TEM mode. Bright-field and high-angle annular dark-field (HAADF) images were recorded. EDX line scans were recorded using an Oxford Instruments X-max 80 silicon drift detector.

### 4.4 Mechanical Analysis of the Sintered Disks

The fracture stress was determined using bending tests that were carried out with a Ball-on-3-Ball (B3B) test setup, as described elsewhere.^57^ The load was increased with a loading rate of 5 N min^–1^ until fracture. Based on the critical failure load *F*, the fracture stress σ_f_ was calculated using eq 1(58)
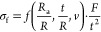
1where *t* is the thickness of the specimen, *v* is the Poisson ratio, *R*_a_ is the support radius, and *R* is the sample radius. The expression  is a dimensionless function, which can be derived using an online tool based on finite element modeling provided by the University of Leoben.^59,60^ Based on the determined fracture stress values, a Weibull statistical analysis was performed using eq 2(61)
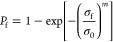
2where *P*_f_ is the failure probability. The characteristic strength σ_0_ is equivalent to the fracture stress for a failure probability of 63.2% and *m* is the Weibull modulus, which describes the variability of the fracture stresses. A linear fitting procedure was implemented for the analysis. Fractographic analyses are required to conclusively determine the dominant failure mechanism.

### 4.5 Cell Assembly and Electrochemical Characterization

For the assembly of a cell, the sintered LATP/LFP/LWO tape was impregnated with a solution of an interpenetrating polymer network electrolyte (IPNE). Details of the synthesis and properties of IPNE are described elsewhere.^39^ In brief, IPNE was prepared using a combination of poly(ethylene oxide) and poly(vinylidene fluoride)-based networked solid polymer electrolytes (O-NSPE and F-NSPE, respectively) containing lithium bis(fluorosulfonyl) imide (LiFSI) in *N*,*N*-dimethylacetamide. Five μL of as-prepared IPNE solution was uniformly dropped onto the top surface of the LFP–LATP/LWO (60:40:5) composite disk using a micropipet at room temperature. The infiltrated electrodes were dried in an oven followed by thermal treatment at 80 °C under vacuum for 4 h to solidify the IPNE and fully remove the residual solvent. The infiltrated cathode was investigated by FIB-SEM followed by image analysis, which indicates a volume fraction of ∼18% IPNE and ∼7% remaining unfilled porosity in the cathode (Figure 6). An ∼100 nm thick Pt layer deposited by sputter coating was used as a cathodic current collector for LFP–LATP composite electrodes.

The IPNE solution was also used to prepare a 70 ± 5 μm thick, free-standing membrane,^39^ exhibiting high ionic conductivity (approximately 1 mS cm^–1^) and high transference number (*t*_Li+_ = 0.69) at 30 °C.

Cell assembly was performed inside an Ar-filled glovebox at room temperature. The IPNE membrane and the Li foil were punched into disks with a diameter of 18 mm. Afterward, the IPNE membrane was placed between an infiltrated LFP–LATP/LWO (60:40:5) composite disk (LFP–LATP, around 75 μm thick with a diameter of 12 mm) and a Li metal anode (∼50 μm thick). The infiltrated electrode/IPNE/Li sandwich was sealed in a CR2032 coin cell for electrochemical measurements. EIS measurements were carried out at the OCV in a frequency range of 100 mHz to 3 MHz with an amplitude of 10 mV at 30 °C by using the EC-lab software V11.43 (Bio-Logic, France). Galvanostatic charge/discharge tests were conducted between 2.8 and 3.9 V (vs Li/Li^+^) at 30 °C using the EC-Lab software V11.32 (Bio-Logic, France), and the applied current density was 50 μA cm^–2^.

## Supporting Information Available

Dilatometer measurement of the shrinking behavior of the LATP–LFP (50:50) mixture and the mixture containing 5 wt % LWO; lattice parameters, refinement statistics of the phases in the investigated samples, with or without LWO additions, XRD pattern of the sintered (*T* = 800 °C) LFP–LATP composite without the LWO additive; XP spectra of Ti^4+^ peaks of LATP/LWO (60:40:5, heated at 700 °C); Mössbauer spectral parameters (mm/s) of LFP–LATP/LWO (60:40:5), recorded at 25 and 800 °C; backscattered electron image and elemental maps of a compressed and not compressed sintered LFP–LATP/LWO; and characteristic strength of the investigated specimens in dependence of the relative density together with its SEM image or from the authors (PDF)
