# Supplementary material for: Enabling High-Performance Hybrid Solid-State Batteries by Improving the Microstructure of Free-Standing LATP/LFP Composite Cathodes
Source: ACS Appl Mater Interfaces. 2024 Apr 1;16(14):17461–73. doi: 10.1021/acsami.3c18542 (PMC11009911; doi:10.1021/acsami.3c18542)
Supplement: Supplementary file 1 — am3c18542_si_001.pdf [file am3c18542_si_001.pdf]

Supporting Information

Enabling high-performance hybrid solid state batteries by improving the microstructure of free-standing LATP/LFP composite cathodes

Martin Ihrig[a,b], Enkhtsetseg Dashjav[a], Philipp Odenwald[a,c], Christian Dellen[a], Daniel Grüner[d], Jürgen Peter Gross[d], Thi Tuyet Hanh Nguyen[e], Yu-Hsing Lin[e], Walter Sebastian Scheld[a], Changhee Lee[e], Ruth Schwaiger[d], Abdelfattah Mahmoud[g], Jürgen Malzbender[d], Olivier Guillon[a], Sven Uhlenbruck[a], Martin Finsterbusch[a], Frank Tietz[a], Hsisheng Teng[e,h,i], Dina Fattakhova-Rohlfing[a,c*]*

[a]Institute of Energy and Climate Research, IEK-1: Materials Synthesis and Processing, Forschungszentrum Jülich GmbH, 52425 Jülich, Germany

[b]Department of Chemical Engineering, National Taiwan University of Science and Technology, No. 43, Keelung Rd., Sec. 4, Da'an Dist. Taipei City 106, Taiwan

[c]Faculty of Engineering and Center for Nanointegration Duisburg-Essen (CENIDE), Universität Duisburg-Essen, Lotharstraße 1, 47057 Duisburg, Germany

[d]Institute of Energy and Climate Research, IEK-2: Microstructure and Properties Forschungszentrum Jülich GmbH, 52425 Jülich, Germany

[e]Graduate School of Engineering, Kyoto University, Nishikyo-ku, Kyoto 615-8510, Japan

[f]Department of Chemical Engineering, National Cheng Kung University, Tainan 70101, Taiwan

[g]GREENMat, CESAM Research Unit, Institute of Chemistry B6, University of Liège, 4000 Liège, Belgium

[h]Hierarchical Green-Energy Materials (Hi-GEM) Research Center, National Cheng Kung University, Tainan 70101, Taiwan

[i]Center of Applied Nanomedicine, National Cheng Kung University, Tainan 70101, Taiwan

Dilatometer measurements

The feasibility of lowering the sintering temperature of LFP-LATP by LWO was studied with pressed pellets by dilatometry. Powder mixtures of LATP and LFP (50:50), and additionally 5 wt% LWO, were uniaxially pressed into pellets with a diameter of 8 mm. The pellets were heated from 30 °C to 1100 °C in a dilatometer (DIL402 C, Netzsch) with a heating rate of 300 °C/h. After reaching the final temperature the samples cooled down with the same rate to room temperature.

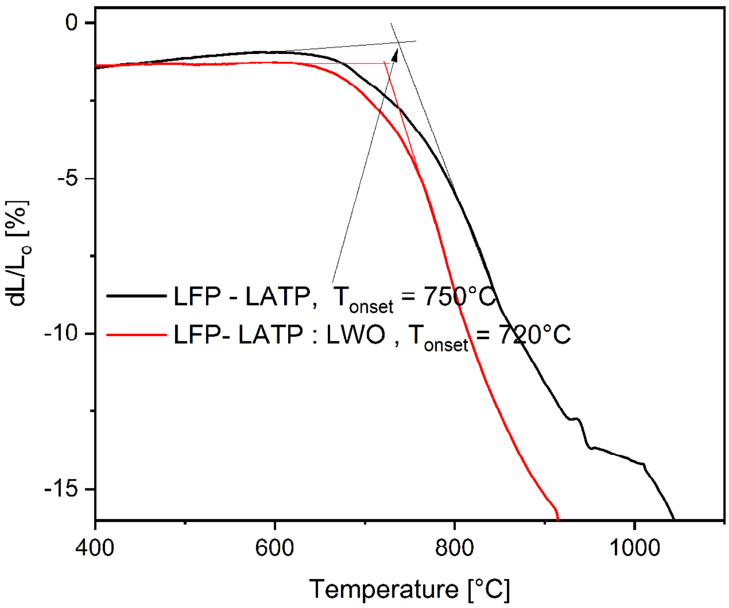

Figure S1. Shrinking curves of the LATP-LFP (50:50) mixture and the mixture containing 5 wt% LWO measured with a heating rate of 300 °C/h in argon .

The dilatometry curves show the shrinkage of the pressed samples with increasing temperature. After an initial small decline in thickness for both samples up to a temperature of 120 °C, the thickness of the LFP-LATP sample (black) increases due to the thermal expansion, while the LFP-LATP-LWO sample (red) continues to shrink. The onset of sintering in both curves, evident by a significant decrease in thickness, occurs at a temperature of 720 °C. The sample with 5 wt% LWO shows a larger shrinkage and, therefore, a higher relative density of the pellet after the measurement This is also evident for pellets sintered at 775 °C for 1 h in argon. The relative densities of such pellets were determined as 72.5 wt%, 84.5wt% for LATP-LFP-C, 5 wt% LWO, respectively. The sintering additive LWO melts at 742°C [1] leading to improved densification in comparison to the samples without LWO and supports liquid phase sintering. However, as the sintering temperature for the tapes is lowered to 720 °C, the melting temperature of LWO is not reached and liquid phase sintering and grain growth was not observed.

XRD analysis

The co-sintering of LFP-LATP (60:40) without LWO addition was performed at 800°C in Ar atmosphere[2] and with LWO was at 700°C. XRD measured and fitted diagram is shown in Figure S2 for the comparison with the sample with LWO additive, as described in main text.

In the Table S1 the lattice parameters and refinement residual values are given for alls samples with and without LWO additives.

Table S1. Lattice parameters, refinement statistics of the phases in the investigated samples, with or without LWO additions

**LFP: LATP +5wt% LWO**	**Phase , SG**	**a [Å]**	**b [Å]**	**c [Å]**	**beta [°]**	**GoF, R_p, R_{wp}**
**40:60**	LFP, Pnma	10.325	6.005	4.692		2.25, 1.83, 2.57
	LATP, Pbna	8.497	8.565	11.859		

	$AlPO_4$, $C222_1$	7.09	7.16	6.99		
	WO_2 ,$P2_1/c$	5.57	4.89	5.66	120.6	
**50:50**	LFP, Pnma	10.326	6.006	4.693		1.76, 1.30, 1.89
	LATP, Pbna	8.494	8.565	11.852		
	$AlPO_4$, $C222_1$	7.01	7.25	6.98		
	WO_2 ,$P2_1/c$	5.58	4.89	5.65	120.6	
**60:40**	LFP, Pnma	10.326	6.006	4.693		1.67, 1.42, 1.96
	LATP, Pbna	8.496	8.565	11.856		
	$AlPO_4$, $C222_1$	7.09	7.16	7.00		
	WO_2 ,$P2_1/c$	5.57	4.89	5.65	120.6	

LFP: LATP without LWO	Phase , SG	a [Å]	b [Å]	c [Å]	beta [°]	GoF, R_p, R_{wp}
	LFP, Pnma	10.326	6.006	4.693		1.55, 1.38, 1.94
	LATP, Pbna	8.503	8.573	11.856		
	$AlPO_4$, C222$_1$	7.162	7.077	6.992		

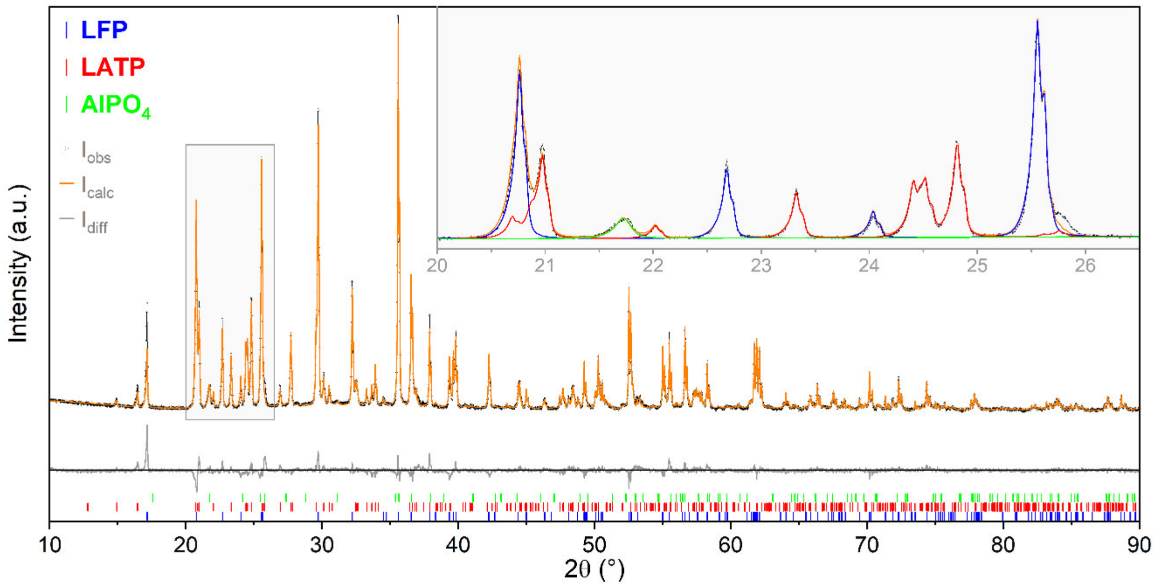

Figure S2. Rietveld analysis of XRD pattern of the sintered LFP-LATP composite without LWO additive. The XRD pattern reveals olivine-type LFP and orthorhombic LATP as main phases, and $AlPO_4$ as a minor phase. The inset provides good visibility of the $AlPO_4$ phase.

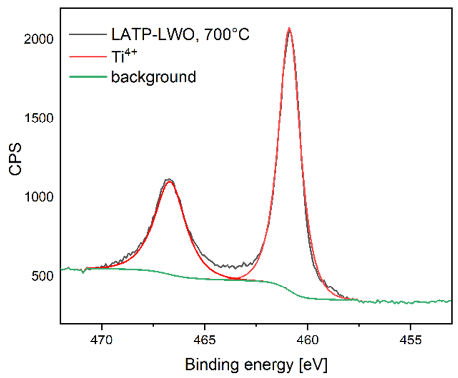

Figure S3. XP spectra of Ti^{4+} peaks of LATP:LWO (60:40:5) heated at 700 °C.

Table S2. Mössbauer spectral parameters (mm/s) of LFP-LATP:LWO (60:40:5) materials recorded at 25 °C and 800 °C.

		**LFP-LATP :LWO 25 °C**	**LFP-LATP: LWO 800°C**
**Fe^{2+} - $LiFePO_4$**	δ (mm/s)	1.22 (1)	1.22 (1)
	Δ (mm/s)	2.95 (2)	2.97 (2)
	Γ (mm/s)	0.28 (1)	0.26 (1)
	Area (%)	93 (1)	97 (1)
**Fe^{3+} - Fe_2P**	δ (mm/s)	0.69 (1)	0.63 (1)
	Δ (mm/s)	0.32 (2)	0.47 (2)
	Γ (mm/s)	0.35 (1)	0.35 (1)
	Area (%)	3 (1)	3 (1)
**Fe^{3+} - in disordered $LiFePO_4$**	δ (mm/s)	0.45 (2)	
	Δ (mm/s)	1.45 (4)	
	Γ (mm/s)	0.26 (1)	
	Area (%)	4 (1)	

a)

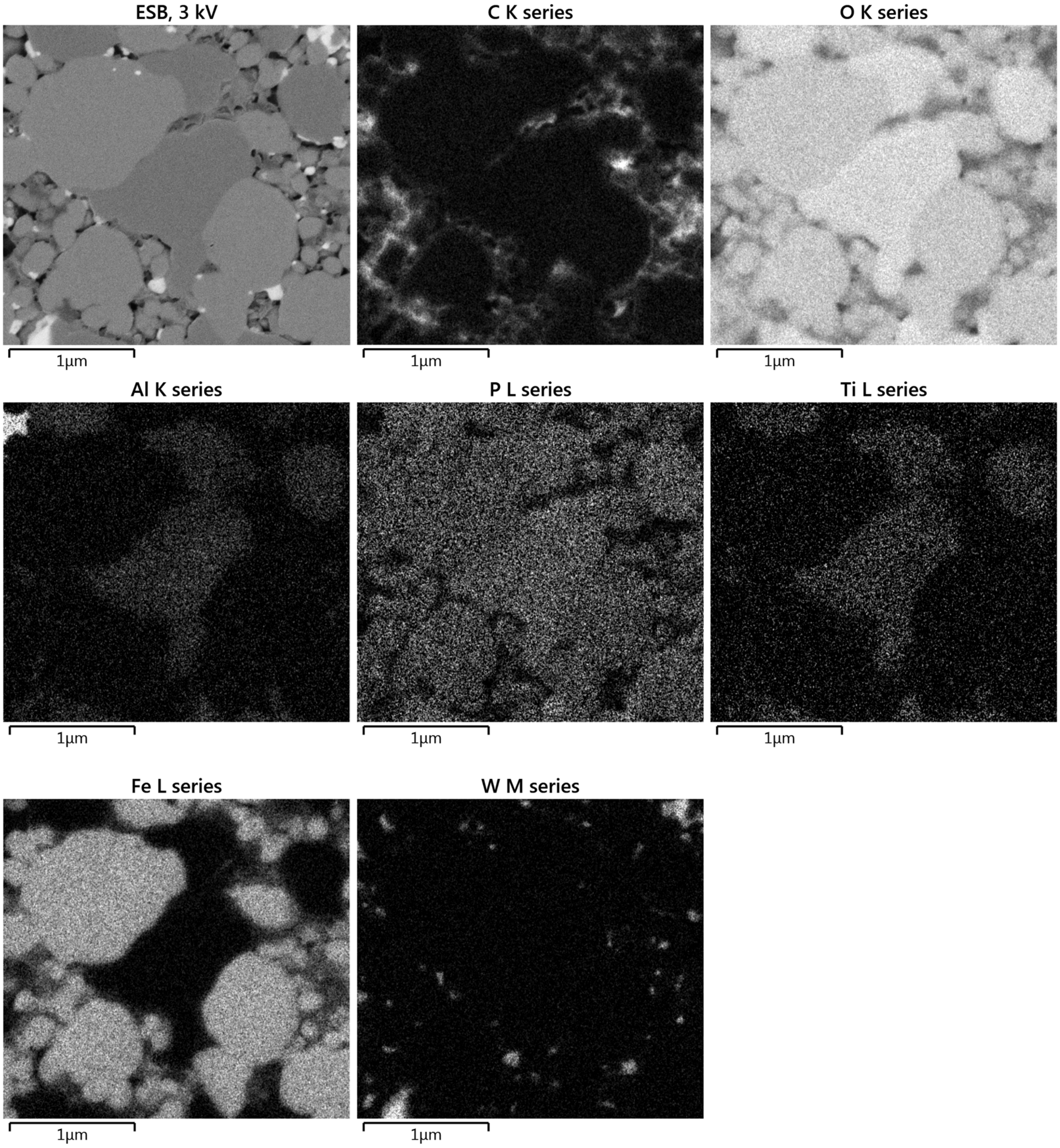
ESB, 3 kV
C K series
O K series
1µm
Al K series
P L series
Ti L series
Fe L series
W M series

b)

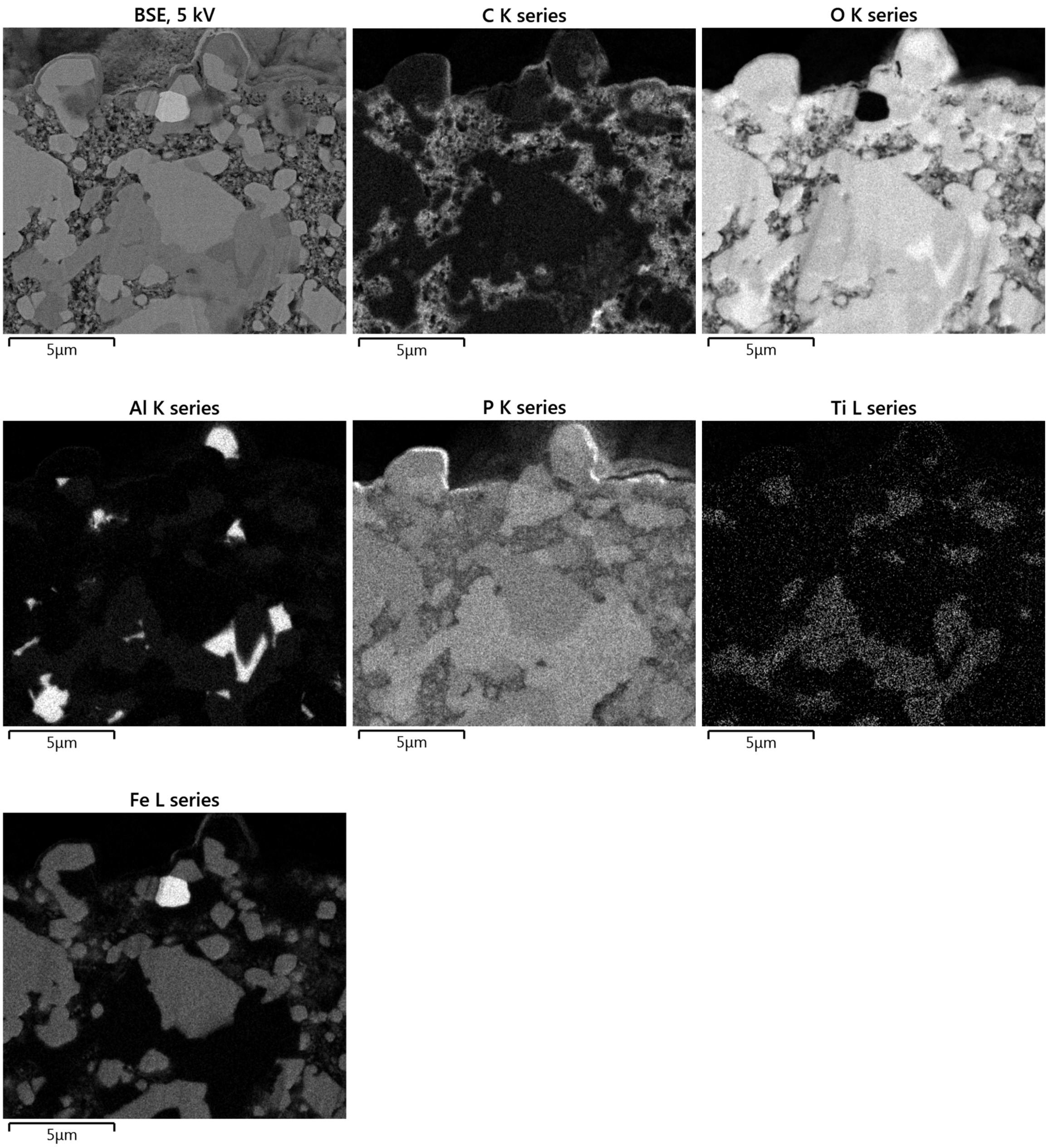

Figure S4. a) Backscattered electron image and EDX elemental maps (3 keV excitation energy) of a polished cross section of a laminated, sintered LFP-LATP:LWO composite. b) Backscattered electron image and EDX elemental maps (5 keV excitation energy) of a polished cross section of a sintered LFP-LATP composite (without LWO).

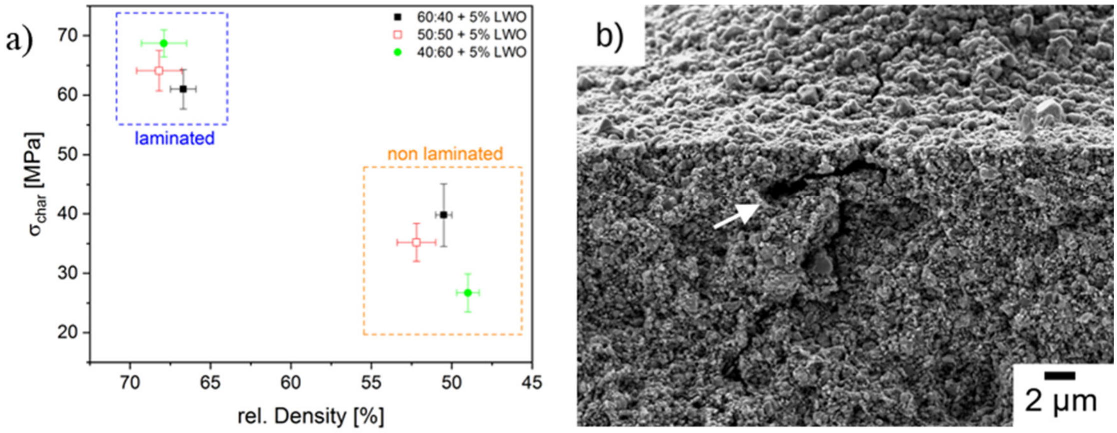

Figure S5. a) Characteristic strength of the investigated specimens in dependence of the relative density. b) SEM micrograph of a fractured sample. The arrow indicates an extended pore near the surface, which is identified as fracture origin.

References

[1] P. Odenwald, Q. Ma, B. Davaasuren, E. Dashjav, F. Tietz, M. Wolff, W. Rheinheimer, S. Uhlenbruck, O. Guillon, D. Fattakhova-Rohlfing, *ChemElectroChem* **2022**, *9*, e202101366.

[2] M. Ihrig, E. Dashjav, A. M. Laptev, R. Ye, D. Grüner, M. Ziegner, P. Odenwald, M. Finsterbusch, F. Tietz, D. Fattakhova-Rohlfing, O. Guillon, *Journal of Power Sources* **2022**, *543*, 231822.
